# The integration of rapid qualitative research in clinical trials: reflections from the ward-based goal-directed fluid therapy (GDFT) in acute pancreatitis feasibility trial

**DOI:** 10.1186/s13063-023-07191-6

**Published:** 2023-03-25

**Authors:** Cecilia Vindrola-Padros, Farid Froghi, Vignesh Gopalan, Sachan Maruthan, Helder Filipe, Margaret McNeil, Sara Mingo Garcia, Brian Davidson, on behalf of the GAP trial team

**Affiliations:** 1 Department of Targeted Intervention, University College London, 3Rd Floor Charles Bell House, 43-45 Foley Street, London, W1W 7TY UK; 2 Division of Surgery & Interventional Sciences, University College London, London, UK; 3 Royal Free London NHS Foundation Trust, London, UK

**Keywords:** Rapid qualitative research, Clinical trials, Acute pancreatitis, Feedback loops

## Abstract

**Background:**

There has been an increase in the integration of qualitative studies in randomised controlled trials. The purpose of this article is to reflect on our experience of carrying out a rapid qualitative study during a feasibility trial of goal-directed fluid therapy (GDFT) in patients with acute pancreatitis, including our sharing of emerging findings and the use of these findings by the trial team.

**Methods:**

The study was designed as a rapid feedback evaluation and combined interviews with staff and patients who took part in the trial.

**Findings:**

The rapid qualitative study pointed to common problems in trial recruitment among multiple sites, where lack of engagement of clinical teams across sites might impact negatively on patient recruitment. The article describes how the use of rapid feedback loops can be used as the trial is ongoing to inform changes in implementation. It also covers the potential challenges of working rapidly and collaborative with the trial team.

**Conclusions:**

Rapid feedback evaluations can be used to generate findings across all stages of trial design and delivery. Additional research is required to explore the implementation of this research design in other settings and trial designs.

## Background

There has been a notable increase in the integration of qualitative studies in randomised controlled trials in healthcare [20, 24]. In part, this is due to the demonstrated value of these studies for determining the acceptability and appropriateness of trials for clinical staff and patients. They are an essential part of establishing barriers to recruitment, improving informed consent and documenting processes crucial to the replicability and implementation of clinical trials across sites and patient groups and informing the interpretation of outcomes [5–7, 20, 25].

The criteria set out for qualitative studies designed as Studies Within A Trial (SWAT) include the following: (1) seek to resolve important uncertainties about the processes used in trials, (2) it is embedded within a host trial, (3) must not affect the scientific integrity of the host trial, its rationale or outcome measures, (4) have a formal protocol, (5) provide data to inform the design and conduct of future trials but will also provide data to inform decisions about the ongoing trial [29]. Qualitative studies are used at different stages of the clinical trial process and in different ways to inform: intervention content and delivery (development, components, underlying theories, benefits, acceptability, feasibility and implementation of the intervention), trial design and implementation (the best time to approach patients, recruitment and retention, participation, acceptability, ethical conduct, adaptation of the trial to the local context and impact of the trial on staff or participants), breadth and variation in outcomes, measures of process and outcome (accuracy and completion of outcome measures) and target condition (experience of disease, behaviours or beliefs in relation to the disease and treatment) [9, 12, 20, 23, 34]. Studies tend to focus on more than one aspect of a trial and often combine multiple methods for the collection of qualitative data, the most frequent being focus groups and interviews [20].

Despite the frequent integration of qualitative research in clinical trials, several authors have recently highlighted that many qualitative studies implemented in this context are labour and time intensive and, as a result, do not produce findings at a time when they can be used to inform decision-making processes relating to trial design and delivery [21]. Additional work needs to be carried out to embed qualitative studies in trials that can produce findings in real-time so these can be used to inform changes in practice [26, 27].

### Rapid qualitative research in clinical trials

Rapid qualitative research is becoming more widely used in healthcare [31]. Rapid data collection and analysis approaches are used to capture complexities surrounding service provision, the social and cultural factors shaping service use and delivery and the nuanced practices of service provision in short time frames, which in turn, can be used to inform and shape implementation and future service design [33]. Common rapid qualitative research approaches include rapid ethnographic assessments (REAs), rapid assessment procedures (RAPs), rapid qualitative inquiry (RQI), rapid ethnographies (including quick, focused and short-term ethnographies) and rapid evaluations (including rapid feedback evaluation, real-time evaluation and rapid assessment). McNall and Foster-Fishman [17] reviewed a wide range of ‘Rapid Evaluation and Appraisal Methods’ (REAM) and proposed an ‘umbrella’ definition:

- Are conducted over a short timeframe (weeks or months)
- Often include participants or members from the community in the phases of study design or implementation
- Combine multiple research methods and triangulate data during data analysis
- Collect data and undertake analysis in parallel, using insights gained from analysis to iteratively shape the data collection process.

We have mapped and critically examined a wide range of rapid qualitative approaches in other publications [13, 30, 32, 33]. Most rapid qualitative studies have been implemented in the context of complex health emergencies [13], to carry out rapid evaluations of interventions or health programmes [32] or in the form of rapid ethnographies in the field of health services research [32, 33].

The most common use of rapid qualitative research in clinical trials is to gather data on the local context or patient population *prior* to the implementation of the trial. For instance, Kitchen et al. [14] carried out a focused ethnography prior to a randomised controlled trial on depression to ensure the setting was adequate for the trial. The study combined 158 h of observation, six formal interviews with staff and the analysis of 17 documents. Data were analysed using thematic analysis. The study found that staff decisions were sometimes based on non-clinical factors such as resource availability. Staff experienced different levels of confidence in relation to making patient diagnoses, but their capacity was shaped by their ability to attend training and integrate learning into practice. The study findings were integrated into the trial protocol [14].

Bond and colleagues [2] carried out a rapid qualitative study before implementing a trial intervention aimed at HIV prevention. The study was carried out over a 2-week period and combined group discussions, interviews and observations to develop a profile (social, demographic and economic) of the sites where the trial implementation team would be working. The data collected for this study were also used early on in the trial to contextualise the results they were obtaining from process indicator data in relation to trial participation [2, 4]. Additional examples include the use of rapid ethnographic assessments to make sure the trial design and information provided to potential participants is culturally sensitive and appropriate [19].

A recent application of rapid qualitative methodology to the implementation for clinical trials is the development of RAPICE by Palinkas, Zatzick and colleagues [21, 35]. RAPICE stands for Rapid Assessment Procedure Informed Clinical Ethnography. It was developed recently to incorporate qualitative research in the field of pragmatic clinical trials by combining structured approaches from Rapid Assessment Procedures (RAP), a rapid qualitative research approach that relies on team-based research and the use of structured data collection methods, with intensive fieldwork commonly used in clinical ethnographies [35].

The aim of RAPICE, and the reason why it is being used in pragmatic clinical trials, is to collect and analyse data on implementation context, process, barriers, facilitators, and perceptions of outcomes. Pragmatic trials are those where the effectiveness of complex interventions is evaluated in real-life conditions (compared to other trials designs that might seek to evaluate interventions in “optimal” conditions) [22]. RAPICE responds to resource restrictions in pragmatic clinical trials by providing findings at a relatively low cost [21].

Similarly, the The QuinteT Recruitment Intervention (QRI) was designed to identify and understand issues with recruitment rapidly by combining multiple qualitative strategies and quantitative data [26]. A recent evaluation of the QRI has demonstrated that it can help improve recruitment in clinical trials by unearthing issues the trial teams were not aware of [8, 27].

REAMs have the potential to inform trial design and delivery in a timely way by running data collection and analysis in parallel and sharing preliminary findings with the trial team on an ongoing basis through previously agreed ‘feedback loops’. Rapid feedback evaluations can be useful for trial teams at all stages of the study, from a predesign stage as described in the studies above, to address informed consent and recruitment issues (as in the QRI) as well as through all stages of trial implementation. In this article, we reflect on our experience of carrying out rapid qualitative research during clinical trials. We use a recent example of a feasibility trial of goal-directed fluid therapy (GDFT) in patients with acute pancreatitis, to reflect on the benefits and limitations of the rapid qualitative design to inform the delivery of the trial. We focus on the research design and methods we used but also include the findings of the study to illustrate the types of findings that were generated and how these were used by the trial team. We also present a proposal for the design of rapid qualitative research in the context of clinical trials guided by the lessons we have learnt within this trial and others.

## Methods

### The GAP trial

There is currently no pharmacological intervention approved for the management of acute pancreatitis, a disease that carries high morbidity and morbidity [18]. The mainstay of treatment has been supportive care with adequate hydration with intravenous fluids. However, there is much debate on the type, timing, volume and rate of fluid therapy. The GAP trial sought to explore the feasibility of delivering fluids using goal directed fluid therapy (GDFT) as guided by a non-invasive cardiac output measurement device on the ward.

A detailed protocol of the GAP trial has been published [10]. In summary, it was designed as a two-centre, feasibility randomised controlled trial. Feasibility was assessed by the ability to recruit patients at the selected sites (recruitment rate), the rate of withdrawal from the GDFT protocol and the reasons for withdrawal from the GDFT protocol. The sample for the trial included a convenience sample of 50 patients, randomised equally to the two groups (GDFT and standard care, which involved fluid administration as deemed appropriate by the clinical team).

Patients were screened by the emergency physicians and trial nurses. Adult patients diagnosed with acute pancreatitis according to the Atlanta criteria [1] were referred to the trial recruitment team within 4 h of diagnosis. They were then approached by the trial nurses and provided with patient information sheets and a discussion about the trial intervention. Consenting adult (> 18 years old) patients were then randomised to GDFT or standard care, and the intervention was started within 6 h of diagnosis of acute pancreatitis.

All patients in the trial had non-invasive cardiac output monitoring every 4 h from the time of randomisation for 48 h. In the case of patients receiving ward-based GDFT, the intervention was carried out for 48 h after randomisation. The GDFT involved optimising the stroke volume (SV, volume pushed out of the heart into the circulation with each beat), following administration of a 250-ml bolus of intravenous fluids. A background infusion of 1.5 ml/kg/hr of intravenous fluids would be started and an initial intravenous fluid bolus was administered to assess the stroke volume (SV) response using the non-invasive monitor attached to the patient’s chest. This process of SV optimisation was repeated every 4 h until the end of the 48-h intervention period. The standard of care group just had the non-invasive monitoring every 4 h without any optimisation.

### The GAP trial rapid qualitative study

The rapid qualitative study was designed in conjunction with the design of the trial and its purpose was to explore the processes used in trial recruitment and delivery as well as staff and patients’ experiences of the trial. As the primary outcome of the GAP trial was feasibility, the rapid qualitative study was also designed to inform future decisions on the potential scale-up and the design of a subsequent multicentre clinical study on cost effectiveness.

The rapid qualitative study had the following aims:

1. Identify the main reasons for individuals declining participation in the trial
2. Identify the reasons that eligible patients have for taking part in the trial
3. Explore patients’ understanding of the trial and their experiences of receiving information relating to the trial
4. Determine the reasons of withdrawal from the trial
5. From the point of view of clinical staff, explore the main issues that hinder recruitment and implementation of the trial
6. Share findings with the trial team on a regular basis so these could be used to inform decisions about trial set-up and implementation

#### Qualitative study design

The GAP qualitative study was informed by a rapid feedback evaluation design [16] where data collection and analysis are carried out in parallel so emerging findings could be shared with the trial team on a regular basis to inform trial design and delivery. These processes of sharing were established in the form of monthly feedback loops where the qualitative researcher shared information on the emerging findings and progress of the qualitative study with the trial team and the trial team considered modifications to the trial logistics to facilitate trial recruitment. The researcher collected data over a period of 3 months (October to December 2019).

The rapid qualitative study consisted of the following:

- (1) semi-structured face to face and telephone interviews with patients recruited to the trial in two sites, (2) semi-structured face to face and telephone interviews with staff involved in the set-up and/or delivery of the trial across two sites, (3) semi-structured interviews with research managers and staff from potential trial sites and (4) a telephone audit (collecting qualitative data) with patients diagnosed with acute pancreatitis who could have taken part in the trial. The interviews were carried out by one researcher (CV) using an interview topic guide and were audio recorded. We have summarised the main components of the qualitative study, including the study sample in Table 1.

**Table 1:** Summary of rapid qualitative study design

Data collection	Sample	Data analysis
Semi-structured interviews with *patients* (audio recorded and interview notes)	7 patients who took part in the trial across both sites (all patients received the intervention). None of the patients who declined participation in the trial accepted our invitation to take part in a telephone interview	Recordings were transcribed and imported into Nvivo. Transcripts were first analysed thematically by using a codebook and a framework was developed
Semi-structured interviews with *staff* (audio recorded and interview notes)	12 members of staff across both sites (including clinical and management staff)	Recordings were transcribed and imported into Nvivo. Transcripts were first analysed thematically by using a codebook and a framework was developed
Audit based on telephone conversations (data collected through notes made on a proforma)	16 potential trial participants (who did not take part in the trial) out of 43 who were approached	Notes were imported into Nvivo, analysed thematically and through framework analysis

#### Interviews

Interviews involved a sample of 9 patients and 12 staff members (clinicians involved in patient care: general surgery registrars, A&E or emergency surgical unit doctors/nurses, clinical nurse specialists, members of research support services). The purpose of the interviews with patients was to explore the following: the reasons that eligible patients had for taking part in the trial, their understanding of the trial and their experiences of receiving information relating to the trial, the reasons that eligible patients had for accepting or refusing the randomisation process and the reasons for withdrawal from the trial. The purpose of the interviews with staff was to explore their experiences with trial set-up, the main issues that hindered recruitment to the study and factors to take into consideration in a potential scale-up of the trial.

#### Recruitment

Patients were handed a qualitative trial patient information sheet by a clinical nurse during their initial GAP trial discussion following hospital admission and were given a brief explanation of the rapid qualitative study. They were given sufficient time (at least 48 h) to accept or decline their involvement in the rapid qualitative research study. They were also given the opportunity to discuss the study with the qualitative researcher. If they agreed to participate, an experienced qualitative researcher then arranged to carry out the interview 24 h prior to planned hospital discharge or by telephone following discharge.

#### Telephone audit

The telephone audit was added to the study after the trial team identified gaps in information related to the reasons why patients declined participation in the trial. It was one of the outcomes of the rapid feedback loops that took place about 2 months after the study began. The audit represented a quick snapshot that could help the team identify the reasons why patients were declining participation in the trial, but it was not meant to be a substitute for the more in-depth information generated through the interviews. Two researchers telephoned all patients who were admitted to the hospital emergency department (ED) and received a diagnosis of acute pancreatitis during the period when the GAP trial was open for recruitment (8th January 2018 for a period of 18 months) but did not agree to take part in the trial. The audit included questions about the care received when the patient attended the ED, their experience of being approached to take part in the trial, the information they received, the reasons why they refused to take part in the trial and their experiences with the management of their symptoms after declining to take part in the trial. The telephone conversations were recorded in the form of structured notes following a pre-established proforma.

#### Data analysis

The interviews were transcribed verbatim. The interview transcripts as well as the notes from the telephone audit were imported into Nvivo. Five initial transcripts were analysed independently by two researchers to familiarise themselves with the data. This early review of the transcripts was used to develop a codebook [15]. The codebook was piloted with three additional transcripts and revised. It was then applied to all of the transcripts and the notes from the audit. One researcher carried out the coding, while a second research cross-checked the codes. After the coding was complete, a framework was developed following the approach for framework analysis proposed by Gale et al. [11].

### Ethical review

Ethics approval was granted by the [blinded] (blinded, project ID: blinded). The trial has been registered on ISRCTN (trial registration number: blinded).

## Results

### Patient perspectives

#### Access to the trial and reasons for not taking part in the trial

Forty-three patients were contacted via telephone to ask about their experiences of being approached for the GAP trial. Out of these 43, only 16 patients agreed to participate in the telephone audit part of the qualitative study as the others were unreachable by phone or indicated that they did not want to answer the questions via telephone.

In the case of the 16 patients who decided to take part in the telephone conversation, 11 (69%) did not remember being approached about the trial and five (31%) remembered being asked about taking part in the trial but declined to take part with the majority (4 out of 5) indicating that this was due to feeling unwell. Severe pain was listed as the main reason for feeling unwell. An additional patient declined to take part in the trial because it seemed “experimental and scary”.

#### Reasons for taking part in the trial

All of the patients who had participated and were interviewed specified that they were interested in taking part in the trial “to help others”. A common belief was that therapies could be tested and improved through the trial, thus benefiting other patients with the same condition.

#### Experiences of going through the trial

All of the patients who were interviewed felt the information provided during the informed consent process was clear. In relation to the delivery of GDFT during the trial, two patients complained about visits made by staff at night for monitoring. It is worth noting that all patients in the trial had cardiac output monitoring and, for half of these patients, this monitoring was used to modify fluid administration. It is also important to mention that frequent observations are part of standard care in acute pancreatitis. The patients interviewed for the study felt that the way this monitoring was done was disruptive for patients who wanted to sleep when in the main ward. A private room was suggested. After discharge from hospital, the trial involved a follow-up telephone call from the trial team. Two patients complained about not knowing when the follow-up calls would happen. They suggested it would be better to book the day and time of the call in advance.

### Staff perspectives

#### Trial design and set-up

Problems were identified with cross-site working when the research nurses were based at only one of the sites. Staff highlighted the need to consider additional research nurse resource, which was necessary to provide night and weekend cover across two hospital sites which are 11 miles apart and 35 min by car. During initial trial set-up, the second hospital which was due to recruit into the study had problems with site set up due to lack of capacity within the Trust: space on wards, ward staff time, research nurse time. The trial PI for the second site attempted to cover additional nurse time through external charity funding, but the hospital responded by reducing the funds for acute nursing staff which exacerbated staff shortages.

There were also concerns that the trial design required 24/7 cover as patients would arrive at unpredictable times and would need to be identified when they presented in the ED. The intensive monitoring of patients on the ward (by the trial team) was also seen as a burden rather than additional support for the ward staff.

#### Patient screening and referral to trial team

Patient screening for the trial was identified as a difficult process as patients presented in the ED and needed to be identified as potential trial participants by doctors in the emergency department. ED doctors then had to flag them to the trial team. In early stages of the trial, there were problems engaging with ED doctors, so the trial team was concerned they might have missed potential trial participants. During later stages of the trial, the trial team developed more robust processes for the identification of patients, such as screening of emergency admission lists, and this facilitated referral of suitable candidates for the trial at the two hospital sites involved in the trial. Following admission, patient care transferred from the emergency department to the surgeons in the surgical admissions team and, hence, a second tranche of doctors had to be informed of the trial and treatment protocol.

Doctor change-over (shifts, locums, holidays, sick leave and rotations) contributed to patients missing the opportunity for recruitment to the trial. The trial team found it is easier to screen patients during regular office hours and to identify potential trial participants at the main trial site (as the team were based there). Trial nurses actively screened ED surgical referrals during office hours for patients with high serum amylase results (diagnostic of acute pancreatitis) and imaging suggestive of pancreatitis. If these were not available, trial nurses screened the referral diagnosis as suspected acute pancreatitis until diagnosis was confirmed. One tool identified by staff as useful for identifying patients in a timely manner was a trial WhatsApp group that brought together staff from both trial sites and the trial team (without disclosing patient identifiable information). Potential patients were flagged and diagnosis was discussed via this group in an anonymised manner.

#### Recruitment

The informed consent process was considered straightforward by staff and patients felt all of the information was clear. According to staff, patients approached at night were less likely to accept to take part. The main reasons for refusal identified by staff were ‘too much discomfort or pain’ and ‘language barriers’.

#### Reasons for withdrawal

According to members from the trial team, the main reasons why patients decided to withdraw from the study included the following: the family did not agree, the patient was discharged prior to 48 h, and the patient was worried about “getting too much fluid”.

#### Follow-up calls to trial participants following discharge from hospital

Staff indicated that it was easier to carry out the follow-up calls during Sunday afternoon as it was a good time to contact people. There were concerns of missing data as some patients did not pick-up the calls.

### Recommendations for trial scale-up

Members of staff made a series of recommendations in relation to the factors that needed to be taken into consideration in the case of a potential trial scale-up. One recommendation was to increase the number of research nurses, so staff could be based at all sites included in trial (the ideal number proposed was 5 nurses for two trial sites). The trial was considered burdensome on staff at stages of patient screening, recruitment and monitoring on the wards, so staffing was frequently mentioned as a key component in future trials. This change in staffing would require changes in funding.

Staff made a series of recommendations to avoid issues with recruitment such as developing strong relationships with clinical teams at all sites before the trial to ensure engagement in referral and recruitment, establishing regular meetings across sites to remind doctors about the trial (and improve referral of potential cases to the trial team), delivering Good Clinical Practice (GCP) training to all doctors involved in the care of acute pancreatitis admissions so they are able to deliver the participant information sheet (PIS) to the patient to avoid unnecessary travel for the research nurse if the patient did not consent to participate in the trial. Staff from the trial team had developed a WhatsApp group to communicate with ED and surgical doctors across both sites and they recommended that this be included in future trials to facilitate early identification of potential trial participants and early mobilisation of trial nursing staff.

Other recommendations had to do with better use of existing staff, such as training research nurses in phlebotomy to draw blood required for trial mechanistic studies at times when the phlebotomist was not available or using clinical nurse specialists (CNS) to sign-post patients and support them throughout the trial.

## Reflections on the use of qualitative findings during and after the trial

In the case of the GAP trial, the qualitative study was designed as an integral component of the trial. The qualitative findings were considered necessary for decision-making processes related to the success of the feasibility trial and the factors that would need to be taken into consideration if the trial was scaled-up. The trial team, however, encountered an issue during early stages of the delivery of the trial, where the qualitative researcher who had designed the study had to leave the team before the qualitative study began. This meant that the qualitative study had to be on hold for a period of time until the team were able to recruit another qualitative researcher.

By the time the second qualitative researcher was able to begin the work, 36 patients had already been recruited to the trial, limiting the available pool of patients who could be approached to take part in the interviews before the trial closed (as the team’s preference was for patients to be approached to take part in the qualitative study when they were in hospital). It also meant that the study had not been able to document staff and patient experiences during early stages of trial set-up and implementation. The second researcher tried to capture these experiences retrospectively through the interviews with staff, but it is possible that important information was missed and changes in trial design that would have improved recruitment in the early stages of trial implementation were not put into practice. It is important to note that despite these delays, the rapid research design allowed the qualitative researcher to complete recruitment for the rapid qualitative study within the originally-established time and budget. Additional work will need to be carried out to determine if qualitative research carried out over short timeframes produces more limited information than longer-term research.

Following a rapid feedback evaluation design, the study involved sharing emerging findings on a monthly basis. As well as sharing study progress and outcomes, the meetings were used for obtaining feedback from the trial team on how to proceed with the study (i.e. how to improve recruitment for interviews and address gaps in data collection, etc.). To facilitate this agile method of frequent knowledge exchange, data collection and analysis were conducted in parallel. This was aided by the qualitative researcher taking extensive notes during the interviews, in addition to the audio recording. After each interview, a summary of the findings was developed and organised into a table (for a detailed description of this approach, see Vindrola-Padros et al. [32]). In addition to these, the table also situated the findings in relation to relevant literature. This table was shared with the trial team to facilitate discussion during the monthly meetings. We have included an example of one of the tables used during early stages of the study in Table 2.

**Table 2:** Example of table used for sharing of findings on a monthly basis with the trial team

Trial area/topic	Main findings	Recommendations from relevant literature
Trial design	• Problems identified with cross-site working and the basing of research nurses at site 1 • Need to consider additional research nurse resource necessary for night and weekend cover and to work at both sites • Difficulties integrating additional sites due to the demand on staff time produced by trial design	
Trial set-up	• No major problems mentioned about set-up at both sites • The problem with set-up at one hospital was mainly due to lack of capacity within the Trust: space on wards, ward staff time, research nurse time, hospital site lead did not see it as a priority	
Patient screening processes	• It is easier to screen patients during office hours • Screening patients from ED seen as a helpful process, but easier to do at site 1	• Trials combining multiple screening approaches (face to face, review of patient lists, through clinical teams) identify higher numbers of potential participants [28]
Referral processes	• Doctors working during night shifts often forget to refer patients • Site 2 experienced delays in referring patients, but this has recently improved • Some doctors do not agree with the trial (i.e. not convinced about evidence), so they will not refer • Doctor change-over led to missing patients	• Receiving frequent feedback from the trial team about trial progress and findings was helpful in maintaining engagement of local clinical teams [3, 9]
Informed consent process	• Considered straightforward by staff • One patient interviewed to date felt all of the information was clear (patient did not know about randomisation, however)	• An ‘open’ informed consent process (allowing the patient to guide the discussion) led to better informed patients [9]
Patient acceptance/refusal	• Patients approached at night were less likely to accept to take part • Main reasons for refusal: patient in too much discomfort/pain, language barriers. Importance of considering effective analgesia prior to being approached for the study	• Trials including an education component for patients about their conditions showed an increase in recruitment rates [3]
Management of patients on the trial	• No problems identified to date	
Patient withdrawal	• Family did not agree • Patient was discharged prior to 48 h (at least 5 patients stayed less than 48 h) • Patient was worried about “getting too much fluid”	
Patient follow-up	• Some patients do not pick-up the calls • More difficult to contact people over the Summer • Sunday afternoon is a good time to contact people	
Factors to consider for trial scale-up and embedding into routine clinical practice	• Increase the number of research nurses, so staff can be based at all sites included in trial (ideal number proposed was 5 nurses for two sites) • Use researcher nurses that live locally to the hospitals to easier to travel at night • Develop strong relationships with clinical teams at all sites before the trial to ensure engagement in referral and recruitment • Establish regular meetings across sites to remind doctors about the trial • Research nurses should be trained in phlebotomy to draw blood at time when phlebotomists are not available • The ‘machine’ should be based close to the nurses’ office (too far away at site 2) • Doctors at all sites receive GCP training early so they can deliver the PIS to the patient to avoid travel for the research nurse from home out of hours or across sites during working hours if the patient is not interested in the trial • The WhatsApp group was seen as a helpful tool • Better use of clinical surgical CNS to sign-post patients • Study can be embedded in critical care • CNS can be involved in supporting patients throughout the trial • The critical care team might have capacity to incorporate this work in routine practice (as they have a large group of trial nurses)	• Training of clinical teams helping with patient referrals has been identified as a useful strategy to increase recruitment [9]

Another important component of these meetings was recording if and how the qualitative study findings were used by the trial team to make changes in the delivery of the trial. In Table 3, we have presented when these were shared, the format used to share them, who was involved in the meetings, and the outcome. We also documented the feedback received from the trial team and how it shaped the qualitative study. As demonstrated in Table 3, the trial team engaged actively with the findings from the study, using them to improve engagement with staff across both trial sites to promote patient recruitment. The feedback meetings were also useful for the qualitative researcher, who received guidance from the trial team on the data that were required to determine the feasibility of the trial and planning required for scale-up.

**Table 3:** Feedback loops in the qualitative study and outcome of the sharing of findings for the trial and the study design

Date meeting	Format	Staff involved	Findings	Use of findings by trial team	Feedback on the study design
October 2019	Summary table	Qualitative researcher, trial team	Identified factors acting as barriers to engage ED staff from second trial site to promote recruitment	Increase communication and reminder messages on WhatsApp group so staff did not forget to flag patients eligible for the trial	Make sure to capture the views of a wide range of staff at both sites, including research managers in charge of trial set-up
November 2019	Summary table	Qualitative researcher, trial team	Confirmed that recruited patients were happy with the way they were approached. Reiterated factors acting as barriers to engage ED staff from second trial site to promote recruitment	Increase communication and reminder messages on WhatsApp group so staff did not forget to flag patients eligible for the trial. Mentioned the trial to ED staff at both sites during meetings and left folder with trial information at both Eds	Capture the views of staff from all hospitals involved in the trial to identify additional issues with trial set-up, delivery and potential scale-up
December 2019	Summary table	Qualitative researcher, trial team	Recruitment barriers identified by staff and patient experiences with recruitment, trial management and follow-up calls	The trial team identified a gap in information regarding the reasons why patients did not take part in the trial (not only those approached, but also those who were not approached)	Include a short telephone audit with patients admitted to the ED to ask about their experiences of being approached for the trial
January 2020	List of main findings to be included in the final report	Qualitative researcher, trial team	List of the areas that will be covered in the final report divided by patient and staff experience and covering issues with recruitment, trial management and potential scale-up	The trial lead proposed organising a workshop with clinical and management staff to discuss these findings before making decisions about the trial scale-up	Include a brief summary of the reasons why patients refused to take part in the trial
February 2020	Final report draft	Qualitative researcher, trial team	Final summary of all qualitative study findings (as presented in this articles)	The recommendations for scale up were used to design a workshop with staff to discuss next steps with the trial and potential plans to scale it up	Feedback was provided to researcher on the report, mainly adding one missing study aim and requesting more information on the sample to be included in the report

## Discussion

We presented the findings of a rapid qualitative study that was integrated in a clinical trial. The rapid qualitative study was used to modify the trial delivery and research pathways within an initial randomised feasibility study and will also aid the design of a subsequent trial aimed at cost effectiveness. The qualitative researcher established monthly feedback sessions with the trial team to deliver emerging findings and seek feedback on the study. These sessions were used to communicate findings on the factors acting as barriers in the recruitment of patients across both trial sites as well as confirm patient satisfaction with the trial recruitment and management processes. The findings were grounded in previous published studies that had identified similar issues and recommendations from the literature were presented to the trial team.

The rapid qualitative study pointed to common problems in trial recruitment among multiple sites, where lack of engagement of clinical teams across sites might impact negatively on patient recruitment. The main reasons for lack of engagement were captured in the interviews with staff and promptly shared with the trial team, so communication could be improved. When sharing emerging findings, the qualitative researcher also highlighted strategies used in other trials to improve staff engagement. These included frequent feedback to local teams about trial progress as well as providing training on trial design and recruitment (including informed consent processes) [3, 9].

The rapid qualitative study also pointed to gaps in data collection as the study was ongoing, where the trial team had not been collecting data on the views and experiences of patients who had refused to take part in the trial. The researcher was able to address this gap in a timely way through a short telephone audit capturing at least some of the views of patients who remembered being approached to take part in the trial but declined and their reasons for doing so. This aspect of the study was important for addressing this gap, but it was also used as a way to generate research capacity in junior doctors without previous experience carrying out qualitative research in a clinical trial.

The study also pointed to issues encountered while implementing the trial that will be useful for informing the potential scale-up of the trial in the near future. Cross-site working was difficult for a small trial team that worked across two sites that were not situated close to each other (11 miles and 35 min by car). The time pressures of the trial made recruitment extremely challenging as is often the case with studies in the emergency setting. Patients with acute pancreatitis would present to the ED at any time of the day or night 24/7 and the window of opportunity for optimising fluid therapy was within the first 24 h after onset. Furthermore, for effective clinical management as part of the trial, the GDFT protocol had to be established within 6 h of diagnosis, which meant that several potential trial participants were not eligible for the trial because of this narrow time window.

It is important to note that the feedback sessions were also helpful for the qualitative researcher, as the trial team provided guidance on the information that was required to complete the trial by the agreed end date. The trial team also made suggestions on the professional groups the researchers could consider approaching. The researcher took these suggestions into consideration, but the process of approaching and interviewing participants was kept separate from the trial team and the anonymity of study participants was maintained throughout the study. The incorporation of the rapid qualitative study was a key factor in the success of the trial completing recruitment on time.

The shaping of trial delivery by the study is an important finding in the sense that many of the documented cases of qualitative research during clinical trials tend to rely on brief stages of research carried out during early stages to inform trial design or in specific periods of trial delivery, such as during the informed consent process [20]. Qualitative research is not always considered as an integral part of clinical trials, as demonstrated in a review of 296 qualitative studies taking place in the context of clinical trials, where O’Cathain and colleagues [20] found that the value of the qualitative research to the trial was not always made explicit. Thanks to their integrated approach, rapid feedback evaluations can help trial teams seeking to implement qualitative studies that can deliver findings across all stages of a trial. In these cases, qualitative research becomes a central component of the trial itself (not an ‘add on’ or temporary stage), facilitating the continuous monitoring of trial delivery and identification of areas for improvement.

## Data Availability

All study data are included in the manuscript.
